# Evaluation of a rapid immunochromatographic test for the detection of low burden *Dirofilaria immitis* (heartworm) in dogs and cats

**DOI:** 10.1007/s00436-017-5709-2

**Published:** 2017-12-09

**Authors:** Marco Genchi, Carlo Mangia, Nicola Ferrari, Sofia Loukeri

**Affiliations:** 1 Department of Veterinary Science, Università degli Studi di Parma, Parma, Italy; 2 Department of Veterinary Medicine, Università degli Studi di Milano, Milan, Italy; 3 BVT-Virbac, 285 avenue de Rome, 83500 La Seyne sur Mer, France

**Keywords:** *Dirofilaria immitis*, Heartworm, Dogs, Cats, Antigen test, Speed Diro™, Sensitivity, Specificity

## Abstract

The performance of a rapid immunochromatographic test for the detection of *Dirofilaria immitis* antigens (Speed Diro™; BVT-Virbac, France) was assessed in 49 experimentally infected dogs and in 244 naturally infected animals; 142 dogs and 102 cats. In experimentally infected dogs, Speed Diro™ showed a sensitivity of 90.9% in dogs infected with one adult female worm and 100% in dogs infected with more than one female worm. Specificity was 100%. For naturally infected dogs, the Knott test and PetChek^®^ HTWM PF served as reference methods for microfilaremia and antigenemia, respectively. All microfilaemic dogs (55/142) were positive with Speed Diro™. Importantly, none of the 21 dogs infected with *D. repens* were positive. The results of Speed Diro™ for the detection of antigenemia were compared with two in-house tests, SNAP^®^ HTWM and Witness^®^ Dirofilaria, and all three tests were 100% specific and sensitive in comparison to PetChek^®^ HTWM PF. For the evaluation of feline samples, 102 cats were examined by echocardiography. Sera from 87 heartworm-infected cats were tested by Speed Diro™ and SNAP^®^ HTWM. The results of Speed Diro™ were equivalent to SNAP^®^ HTWM, with a sensitivity of 98.9% and a specificity of 100%.

## Introduction

Canine heartworm disease (HWD) is a severe and potentially fatal disease worldwide and is endemic in many countries, caused by *Dirofilaria immitis*, a vector-borne nematode*.* Adult parasites are located in the pulmonary arteries and occasionally in the right heart ventricle of infected dogs. The parasite is transmitted to dogs by mosquitoes and infective larvae mature and migrate through the body over several months before reaching the pulmonary arteries, where they mature to the adult stage. Sexually mature females release first stage larvae (microfilariae) into the blood stream. Mosquitoes become infected during a blood meal from microfilaraemic dog (McCall et al. 2008). The pathological response of heartworm infection is mainly due to endarteritis caused by the adult worms in the pulmonary arteries, complicated by perivascular inflammation, caused by the presence of *Wolbachia (*the bacterial endosymbiont of filarial worms)*. Wolbachia*-derived molecules act as pathogen-associated molecular patterns when the parasites die (Kramer and Genchi 2014).

Infection can be diagnosed by the presence of microfilariae in fresh blood samples, with techniques including the modified Knott test (Knott 1939), filtration, and histochemical staining of microfilariae (reviewed by McCall et al. 2008). However, “occult” infections, i.e., without circulating microfilariae, can occur (McCall et al. 2008).

Serological testing for circulating antigens of adult female worms has been available since the late 1980s, carried out either by veterinary reference laboratories as well as in-clinic test kits (Thilsted et al. 1987; Brunner et al. 1988). These tests detect the presence of adult female worms. Currently, ELISA-based and lateral flow immunochromatographic test kits are available and considered highly sensitive and specific methods for infection screening or for confirming a clinical suspicion, further allowing the detection of occult filariosis. However, false-negative results can occur with low worm burdens and/or in cases of low antigenemia (American Heartworm Society 2014).

Cats can be also infected by *D. immitis* and the prevalence of the disease in cats represents 5 to 20% of the prevalence in dogs from the same geographic area (Ryan and Newcomb 1995; Hermesmeyer et al. 2000). Cats are considered a susceptible, but not ideal, host for *D. immitis*. This is reflected by the prolonged pre-patent period, the low number and short life span of adult worms, and the low level and short duration of microfilaremia. Clinical signs of heartworm infection in cats are very different compared to dogs. Clinical and subclinical manifestations of feline dirofilariosis are associated with the migration and death of immature worms or the presence of mature worms in the pulmonary arteries which leads to an inflammatory response, the so-called Heartworm-Associated Respiratory Disease (HARD) (European Society of Dirofilariosis and Angiostrongylosis 2017).

The aim of this study was to assess the performance of a rapid immunochromatographic test kit for the detection of *D. immitis* circulating antigens (Speed Diro™; BVT-Virbac, France) in sera from dogs with a known number of adult worms and in field conditions in sera of dogs and cats living in endemic areas for heartworm infection in Northern and Central Italy. The test kit has recently been modified to improve the readability of the results. The performance of the previous kit was equally good, with a sensitivity and a specificity of 98.7 and 100%, respectively (Genchi et al. 2013) but improved readability of the new test permits better and easier interpretation. A double filter pad has been integrated that improves the filtration of the sample, especially when using whole blood or samples containing residual blood components (cells, hemoglobin), thus limiting non-specific detection. Furthermore, a new hypertonic reagent has been included to increase the readability of the reaction bands.

## Material and methods

Forty-nine sera from experimentally infected dogs (supplied by TRS Labs Inc., Athens, GA, USA) with a known number of adult worms and 48 sera from dogs aged ≤ 5 months (negative control), were included in the study. Dogs were infected by intravenous transplant of adult parasites (8 to 28 months old) and the period between worm transplantation and blood sampling was 1 month. A further 142 dog sera and 102 cat sera were sampled from animals living in endemic areas for heartworm infection (North and Central Italy) using a non-probabilistic sampling procedure of convenience.

Sera from experimentally infected dogs and control dogs were examined by Speed Diro™. All blood samples from the naturally infected dogs (142) were examined using the Knott test for circulating microfilariae and their sera were examined by Speed Diro™ and by other rapid, in-clinic test kits for comparison (two ELISA-based test kits: SNAP^®^ HTWM, IDEXX Laboratories Inc. and PetChek^®^ HTWM PF, and one rapid immuno-migrating test: Witness^®^ Dirofilaria, Symbiotics). All analyses were performed in duplicate and according to the manufacturer’s instructions. For PetChek^®^ HTWM PF, the laboratory procedure was followed and assumed as a reference test. All cats were examined by echocardiography to assess the presence of heartworm infection (the sensitivity of this diagnostic method is reported to be between 88 and 100%; DeFrancesco et al. 2001; Venco et al. 2015), and their sera were examined by Speed Diro™ and SNAP^®^ HTWM (IDEXX Laboratories Inc.).

### Statistical analysis

The sensitivity and specificity of the rapid, in-clinic tests were estimated, with a 95% of confidence interval, considering as positive dogs experimentally infected and, as negative, control dogs ≤ 5 months of age. For cats, echocardiography was considered as the reference method.

## Results

The number of adult worms (male worms and female worms) found in experimentally infected dogs is presented in Table 1. Twenty-five dogs were infected by female worms only. Eight dogs had one adult female worm, seven had two adult female worms, nine dogs had three adult female worms, and one had seven female worms. Twenty-four dogs had mixed infections (female and male worms). The number of adult male worms varied from three to 25. The Speed Diro™ test gave positive results in ten out of 11 dogs infected with only one female worm. All the dogs infected with two or more adult female worms reacted positively to the Speed Diro™, regardless of the presence of the adult male worms (Table 1). No positive reaction was seen in the control dogs. Overall, the estimated sensitivity was 98.0% (95% C.I. 89.2–100.0) and specificity 100% (95%, C.I. 92–100). Considering the number of adult female worm infections, the sensitivity was 90.9% (95% C.I. 58.7–99.7) in dogs infected with one adult female worm, and 100% (95% C.I. 91.4–100) in dogs infected with more than one adult female worm.

**Table 1:** Performances of Speed Diro™ test kit in 49 sera from experimentally *Dirofilaria immitis*-infected dogs with known number of adult worms

Examined dogs	Total number of adult worms	Only female worms no. of worms (no. of dogs)	Female and male worms no. of worms (no. of dogs)	Speed Diro™ positive samples
8	1	1F (8)	(0)	7
10	4	2F (7)	1F + 1 M (3)	10
10	6	3F (9)	2F + 1 M (1)	10
1	4	(0)	2F + 2 M (1)	1
1	7	7F (1)	(0)	1
8	6	(0)	3F + 3 M (8)	8
8	16	(0)	6F + 10 M (8)	8
3	40	(0)	15F + 25 M (3)	3

*F* female worms, *M* male worms

Of the 142 canine blood samples examined by Knott, 66 were microfilaria negative, 21 *D. repens* microfilaria-positive, and 55 *D. immitis* microfilaria-positive. Of the 66 microfilaria negative-sera, 42 (63.7%, 95% C.I. 54.5–72.0) were positive with Speed Diro™, SNAP^®^ HTWM and Witness^®^ Dirofilaria tests. The positive results were confirmed with PetChek^®^ HTWM PF, demonstrating that antigen tests identified an important proportion of occult heartworm infections. None of the 21 *D. repens-*infected dog’s sera reacted positively to any tested kits, showing no diagnostic difference between them in terms of specificity. From 55 sera of *D. immitis* microfilaraemic dogs, all were found positive to all heartworm test kits. Speed Diro™, SNAP^®^ HTWM, and Witness^®^ Dirofilaria demonstrated 100% specificity (95% C.I. 92.13–100) and 100% sensitivity (95% C.I. 96.19–100) versus PetChek^®^ HTWM PF.

From 102 cats, 15 were found to be negative and 87 positive at echocardiography. All negative cats were found to be negative both to SNAP^®^ HTWM and Speed Diro™ kits. From 87 echo-positive cats, 86 were found to be positive both to Speed Diro™ and SNAP^®^ HTWM PF (sensitivity 98.9%, 95% C.I. 93.8–99.9; specificity 100%, 95% C.I. 78–100).

## Discussion

Currently available antigen test kits are able to detect circulating antigens released by female worms in infections consisting of at least one mature female worm and are nearly 100% specific and highly sensitive (Atkins 2003; Courtney and Zeng 2001; McCall et al. 2001; Lee et al. 2011). In our study in experimentally infected dogs, eight dogs had only one adult female worm and three dogs had one female worm and one male worm. Of 11 dogs infected by one female heartworm, 10 reacted positively to Speed Diro™ test kit (90.9%). All dogs infected by two or more female worms were found to be positive with this test kit.

Regarding the data obtained from sera of naturally infected dogs, both microfilaraemic and non-microfilaraemic, all rapid, in-clinic tests presented 100% sensitivity and specificity in comparison to PetChek^®^ HTWM PF which was used as the reference method. The results of the present study confirm the previous data from Vezzani et al. (2008), in which Speed Diro™ results were identical to the results obtained by Witness^®^ Dirofilaria and SNAP^®^ HTWM in 113 *D. immitis* microfilaraemic dogs. None of the heartworm antigen detecting tests showed false-positivity in sera from *D. repens*-infected dogs.

In conclusion, Speed Diro™ was highly sensitive in experimentally infected dogs with low worm burdens. In naturally infected dogs, Speed Diro™ was 100% sensitive and specific, demonstrating equivalent performances to the other rapid, in-clinic tests (SNAP^®^ HTWM and Witness^®^ Dirofilaria) and confirmed by PetChek^®^ HTWM PF, used as the reference method.

The new hypertonic reagent of Speed Diro™ allowed a good washing of the membrane through synergic action between detergents and salts. The results of the test band were easily readable on a white background and no doubtful results were observed.
